# Flat Plasmonic Biosensor with an On-Chip Metagrating-Integrated Laser

**DOI:** 10.1021/acssensors.5c01997

**Published:** 2025-10-08

**Authors:** Erik Strandberg, Mindaugas Juodėnas, Hana Šípová-Jungová, Mikael Käll

**Affiliations:** † Department of Microtechnology and Nanoscience, Chalmers University of Technology, 412 96 Gothenburg, Sweden; ‡ Institute of Materials Science, 70309 Kaunas University of Technology, 514 23 Kaunas, Lithuania; § Department of Physics, Chalmers University of Technology, 412 96 Gothenburg, Sweden

**Keywords:** metasurface, vertical-cavity surface-emitting laser, surface plasmon resonance, compact biosensing, lab-on-a-chip

## Abstract

Metasurfaces are emerging as a transformative platform in optics, offering compact and versatile alternatives to bulky traditional components. Here, we present a metasurface-enabled, on-chip surface plasmon resonance (SPR) biosensor that enables label-free biomolecular analysis in a miniaturized, chip-scale format. By integrating flat metaoptics together with semiconductor lasers, a collimated fan of light for angle-resolved SPR measurements can be emitted directly into a glass substrate, eliminating the need for conventional optics. As a proof of concept, we demonstrate a bulk refractive index sensitivity of 4.9 × 10^–6^ RIU, along with multiplexed detection of low-molecular-weight microRNA with limits of detection of 0.1 nM via direct sensing and 0.02 nM with antibody amplification. The scalability of metasurface fabrication, coupled with the low cost of semiconductor lasers, suggests this platform can be readily adapted for diverse sensing modalities and mass manufacture, potentially transforming SPR beyond its conventional, benchtop implementations.

## Graphical abstract

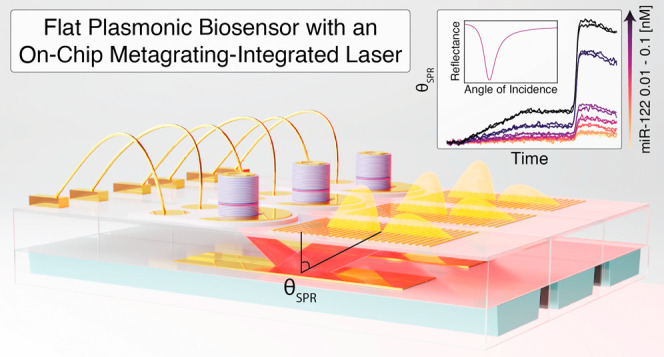

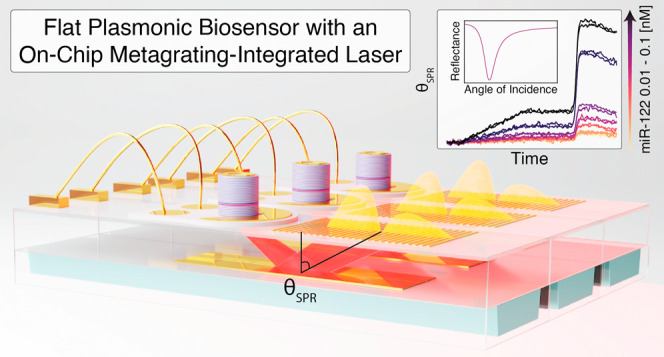

Numerous biosensing methodologies and devices have been developed to meet the ever-increasing demand for sensitive, rapid, and cost-effective biomolecular analysis. Among these, label-free biosensors are crucial because they offer the possibility of real-time monitoring of biomolecular properties and reaction kinetics. These advantages are best exemplified by the surface plasmon resonance (SPR) biosensor, which has been the most used label-free biosensing technology since its commercialization in the 1990s.^1–4^ However, because of high costs, complexity, and bulky nature, SPR instrumentation is still largely restricted to high-end laboratories in academia and industry.^5^ This has motivated significant research efforts targeting affordable, miniaturized, and user-friendly SPR devices that could broaden the user community and open up a wider range of applications.^6^ These efforts have led to a variety of compact SPR systems utilizing technologies such as light-emitting diodes,^7,8^ grating couplers,^9^ optical fibers,^10,11^ and even smartphones as the light source and detector.^12^ In parallel, researchers have also developed a broad range of localized surface plasmon resonance (LSPR) sensors, which instead rely on metal nanoparticles and nanostructures to achieve label-free sensing at a potentially lower cost and smaller footprint.^13^ However, despite these advances, a clear gap still remains between laboratory-scale devices and the availability of low-cost, portable, and robust SPR platforms. Many proposed systems either compromise sensitivity, require complex fabrication, or lack full integration for user-friendly operation. Bridging this gap is essential to unlock the broader potential of label-free biosensing in real-world, point-of-need settings.

Over the past several years, optical metasurfaces have emerged as one of the most dynamic and fast-growing fields of photonics.^14^ Based on 2D arrays of nanostructures, metaoptics shape light by imparting a designed spatial phase distribution on a transmitted or reflected light wave. This enables a drastic reduction of the size and weight of optical components, since optical functions can be encoded within a single subwavelength thick layer.^15^ Moreover, integration of metaoptics with vertical-cavity surface-emitting lasers (VCSELs) directly “on-chip” produces a light source that can emit an arbitrary beam suitable for a wide range of applications. In such a device, a metasurface is etched directly into the facet of a VCSEL,^16,17^ one of the most common types of semiconductor lasers due to its high efficiency, circular beam emission, high fabrication yields, and outstanding high-speed performance.^18,19^ The resulting devices are known as metalasers, which combine coherent emission with spatial beam shaping, offering extremely compact and customizable light sources that hold potential to miniaturize a broad range of applications.^20–22^ In this work, we utilize this concept to demonstrate an essentially “flat” SPR biosensor, as schematically illustrated in Figure 1.

**Figure 1:**
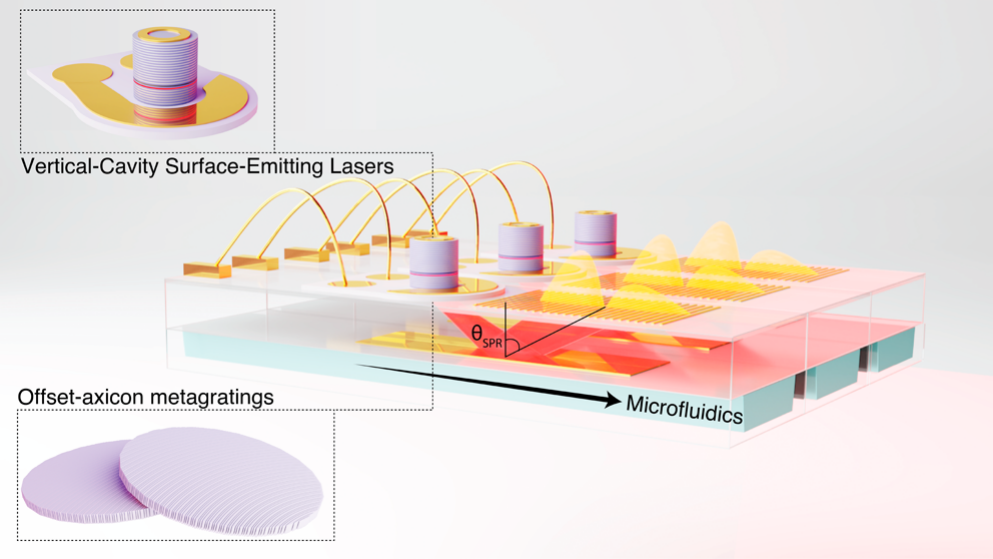
Schematic of the miniaturized planar SPR module. The illumination for the module is provided by a metalaser array, an array of VCSELs with monolithically integrated offset-axicon metagratings, bonded to a glass slide. Each laser in the array can emit a collimated fan-shaped beam toward continuous gold sensing strips on the opposite surface of a thin glass wafer. Surface plasmons are excited at the gold–solution interface, and the reflected intensity distributionis captured by a camera to determine the SPR excitation angle through spatial analysis. The dimensions of the sensor with three individual channels are 2.6 × 2.6 × 1 cm (Figure S1).

Our device is based on launching surface plasmons on a 50 nm thick continuous gold film in the well-known Kretschmann configuration,^23^ which is the most widely used and arguably the most reliable implementation of SPR. However, in contrast to the classical setup, which utilizes an external laser, focusing optics, and a bulky coupling prism to meet the plasmon excitation condition, our device directly produces a fan of light with the range of angles needed for angle-resolved SPR. With both the coupling optics and the light source fabricated in a single semiconductor chip, surface plasmons can be excited in a fully planar, miniaturized, and scalable solid-state SPR module. Moreover, due to the small size of the components, several independent SPR sensing channels can be realized with a single chip. By including microfluidics, multiplexed SPR biosensing experiments can be performed with a standard camera for read-out of the SPR signal. Our device is compact and affordable, does not require alignment, and shows a refractive index resolution of *R* = 4.9 × 10^–6^ refractive index units (RIUs), sufficient for most biosensing tasks. We characterize the device performance,^24^ including its bulk sensitivity using glycerol solutions and its thin-layer sensitivity using a model protein-binding reaction. Finally, we quantify the limit of detection (LoD) using micro-RNA binding to a specific complementary DNA.^25,26^ We believe that the presented concept provides a foundation for accessible, affordable, and extremely compact SPR sensors with wide application potential, including integrated lab-on-a-chip sensor systems, point-of-care biomedical analysis devices, and hand-held sensor units for environmental or biochemical monitoring in the field.

## Results

### On-Chip Illumination Source

The light source for SPR excitation is a bottom-emitting, oxide-confined GaAs VCSEL with a lasing wavelength of λ = 984 nm (Figure 2A). The InGaAs quantum wells that provide the required optical gain are sandwiched between two distributed Bragg reflectors grown on a 600 μm thick GaAs substrate. The emitted linearly polarized Gaussian beam has a significant divergence due to the small oxide aperture (diameter 2 μm), resulting in a beam width of 88 μm (1/e^2^ width) at the bottom outcoupling GaAs facet. The VCSELs were characterized without an integrated metasurface to verify their functionality (Figure 2B), yielding a lasing threshold current at *I*_th_ = 0.3 mA and a maximum output power of *P*_max_ = 2.44 mW. Figure 2C summarizes the single-mode output spectra at various driving currents. The spectrum demonstrates the small red-shift in lasing wavelength with increasing current, due to the thermal build-up in the laser cavity.

**Figure 2:**
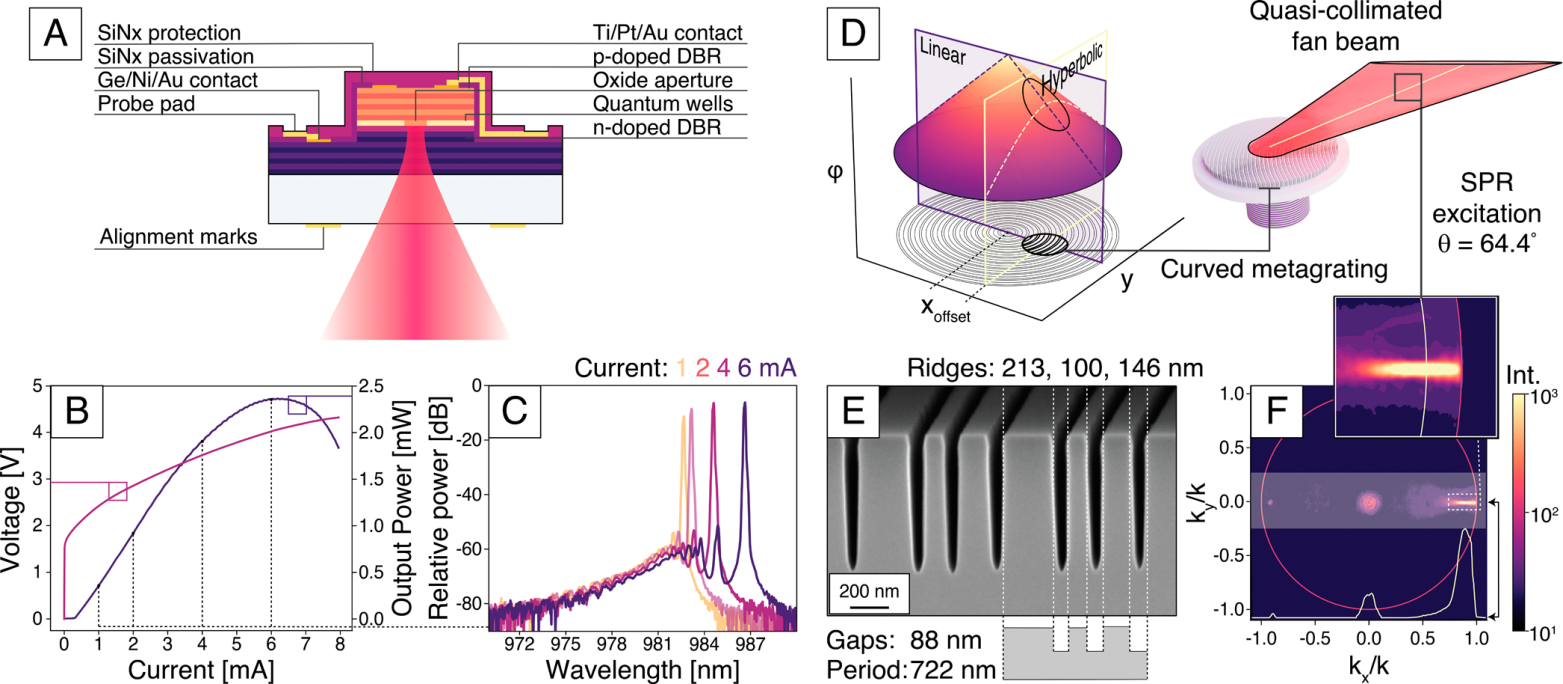
Description of the VCSEL and the integrated offset-axicon metagrating. (A) Cross-sectional schematic of the fabricated bottom-emitting, single-mode VCSEL. (B) Current–power–voltage (IPV) characteristics for asingle-mode VCSEL with an oxide aperture diameter of 2 μm, measured without the integrated metasurface. (C) Emission spectra of a single-mode VCSEL at various injection currents. The VCSEL is lasing in a single transverse and a single longitudinal mode, with a resolution-limited linewidth Δλ < 0.2 nm. (D) Schematic of the offset-axicon metasurface. The metasurface is composed of concentric ring-like metagratings engineered to replicate the conical phase profile of an axicon. By introducing a lateral offset in the phase distribution along the *x*-axis, a combined in-plane linear and out-of-plane hyperbolic phase gradient is achieved. This configuration generates a directional fan-shaped beam centered at an emission angle of 64.01°, optimized to span the angular range relevant for SPR sensing in aqueous environments (*n* = 1.33–1.34). (E) SEM cross-sectional image of a fabricated metagrating. (F) Fourier-plane image with the angular emission profile of the metalaser. The inset highlights the +1 diffraction order, with a profile that corresponds to the fan-shaped beam that is used for the angle-resolved SPR measurements. Note the logarithmic intensity scale.

To shape the emitted beam from the VCSEL to cover the range of angles needed to excite surface plasmons on a gold-water interface from glass, an offset-axicon metagrating^22^ is directly etched into the GaAs substrate (Figure S2). The design process for the metasurface starts by optimizing a supercell metagrating for high-angle deflection (Figure S3). This metagrating is arranged in concentric rings, effectively transforming the phase profile into an axicon with a conical phase profile, as shown in Figure 2D. A conical phase profile would shape a Bessel beam if it were aligned with the VCSEL aperture. However, by shifting the axicon profile off-center along the *x*-axis (Figure 2D), the phase distribution will instead be linear along the *x*-axis and hyperbolic along the *y*-axis. With an x-offset of 240 μm (eq S1) the hyperbolic phase will collimate the diverging Gaussian wavefront emitted by the VCSEL along the *y*-axis, while along the *x*-axis the divergence is maintained but centered around a deflection angle defined by the periodicity of the metagrating. The result is a quasi-collimated fan of light with an angular spread that enables the angle-resolved SPR experiments.

The offset-axicon metagrating was monolithically integrated within the GaAs substrate of the bottom-emitting VCSEL. This approach leverages the high crystalline quality of the GaAs material, enabling the fabrication of a high-efficiency metagrating while ensuring inherent alignment with the laser source (see Supplementary Section 1 for metalaser fabrication). Figure 2E shows a cross-section of the fabricated metagrating. The metagrating is composed of supercells with a periodicity of *P* = 722 nm, designed to direct light into the first diffraction order (*T*_+1_) at an angle of 64.01° within the glass substrate (*n*_glass_ = 1.51). This angular deflection aligns with the SPR excitation conditions at a gold–sample interface for typical sample refractive indices in the range *n* = 1.33–1.34. The trench dimensions within each supercell are optimized for efficient energy transfer into *T*_+1_, following the design principles in ref 22. To ensure compatibility with monolithic fabrication, the optimization was performed with the constraint of equal trench widths, which circumvents the aspect ratio-dependent etching (ARDE) associated with monolithic integration. The emission profile of the metalaser is characterized using Fourier imaging with a high numerical aperture, NA = 1.49, oil-immersion objective (Figure 2F and see Figure S4 for the Fourier imaging setup). The profile corresponds to a quasi-collimated fan-shaped beam, since it can be seen to have a large angular spread along the *x*-axis and a very small spread along the *y*-axis, which closely follows the simulated offset-axicon metagrating design.^22^ The measured relative transmission efficiency was found to be η_rel_ = 0.71, which is in good agreement with the simulated relative efficiency, η_rel_ = 0.76 (see Supplementary Section 2 for a description of the metagrating design and characterization).

### Sensor Design and Operational Principle

Figure 3 schematically illustrates the sensor and its working principle. The full device consists of two parts: the sensor and the SPR chip, implemented as two glass slides joined via index-matching oil to allow for easy disassembly. The sensor slide supports the metalaser array, which is bonded to the glass surface using an index-matched optical adhesive. Because of the flat configuration of the device, the light reflected from the gold film retains the incident in-plane momentum and continues to be totally internally reflected at the top glass surface of the sensor slide. To enable readout from the top surface, the sensor slide is patterned with a periodic array of 2 μm wide gold lines spaced 15 μm apart. The gold lines discretely probe the reflected intensity distribution with an angular sampling rate of ≈ 0.1°. The SPR chip is patterned with metal stripes consisting of a 2 nm titanium adhesion layer and 50 nm of gold, that supports the surface plasmon polaritons, and bonded with PDMS microfluidic channels for delivering various samples (Figure S5). The full sensor in this study utilized three independent metalasers, probing three separate microfluidic channels with individual inlets and outlets. The channels have equal lengths and number of bends to ensure equal hydraulic resistance, and they are fed by an external peristaltic pump (see Supplementary Section 3 for the fabrication of the SPR chip).

**Figure 3:**
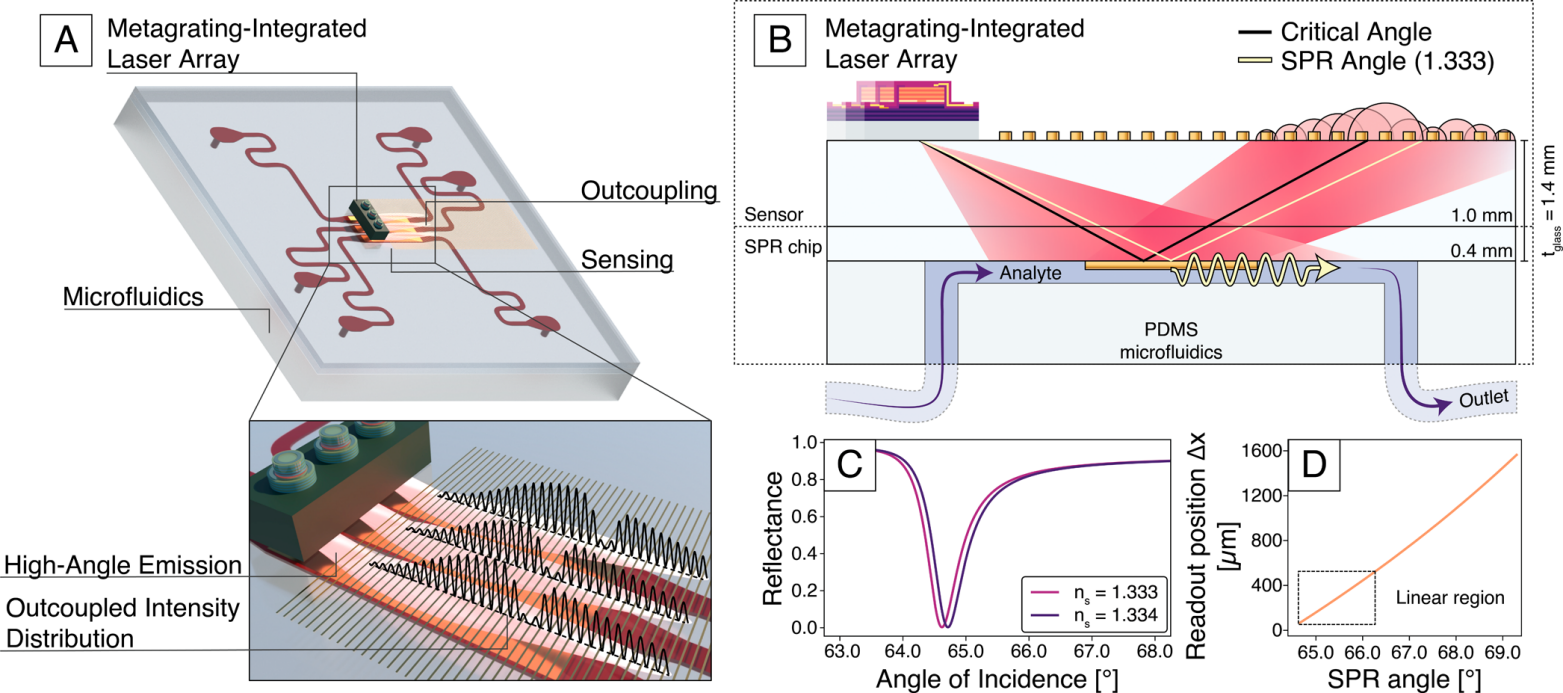
Design and working principle of the flat miniature SPR sensor. (A) Schematic of the sensor, showing the metalaser array and integrated PDMS microfluidic channels. The inset highlights the spatial distribution of outcoupled reflected intensity at the device’s top interface. (B) Cross-section of the SPR sensor structure: A metalaser with a monolithically integrated metagrating illuminates a 50 nm gold film in contact with the liquid in the microfluidic channel. Surface plasmons are excited at the gold–liquid interface, and the reflected light is coupled out by an array of gold lines at the top surface. The SPR excitation angle is extracted from the spatial intensity distribution of the outcoupled light. (C) Simulated reflectance of the gold film as a function of incidence angle for two sample refractive indices: pure water (*n*_s_ = 1.333) and water with 1*%* glycerol (*n*_s_ = 1.334). (D) Conversion from spatial readout position to SPR angle based on the planar configuration in panel (B), as described by eq 2. The conversion is highly linear for small shifts in the SPR angle, enabling sensitive detection of refractive index changes.

For homogeneous media, momentum matching yields the SPR condition as^4^

$$\sin\;\theta_{\mathrm{SPR}}=\frac{1}{n_{\mathrm{glass}}}\sqrt{\frac{|\varepsilon_{\mathrm{Au}}|n_{s}^{2}}{|\varepsilon_{\mathrm{Au}}|-n_{s}^{2}}}$$ 1

where *n*_glass_ = 1.51 is the refractive index of the glass substrate, ε_Au_ = −40.6 + *i*2.22 is the complex permittivity of gold at the laser emission wavelength,^27^ and *n*_s_ is the refractive index of the medium within the SPR evanescent field in the sensing channel. As an example, the analytical angular reflectance spectra in Figure 3C indicate that a change in refractive index from *n*_s_ = 1.333 (pure water at room temperature) to *n*_s_ = 1.334 (water with 1% glycerol) will give a shift in SPR angle of 0.1° (see Figure S6 and Supplementary Section 4). In our setup, the SPR angle is obtained from the lateral position of the reflected intensity minimum, *x*_SPR_, relative to the metalaser source:

$$\theta_{\mathrm{SPR}}=\arctan\left(\frac{x_{\mathrm{SPR}}}{2t_{\mathrm{glass}}}\right)$$ 2

where *t*_glass_ = 1.4 mm is the total thickness of the glass substrate (Figure 3B). For small refractive index variations, *x*_SPR_ changes approximately linearly with θ_SPR_, as presented in Figure 3D.

### Bulk Refractive Index Sensitivity and Resolution

Figure 4A shows a representative reflected intensity distribution on the chip readout area (see Figure S8 for the experimental setup). The distribution is a product of the Gaussian intensity profile of the VCSEL, the angular variation in gold film reflectance, and a slightly angle-dependent scattering of the gold readout lines (Figures S9 and S10). Since all light emitted from the metalaser is confined within the sensor glass slides, some interference from spurious internal reflections is present in the detected reflectance. However, the intensity dip due to SPR excitation is clearly the dominant feature in the spectrum, and it is in good agreement with a calculated reflection spectrum for a 50 nm gold film in contact with pure water *n*_s_ = 1.333 (see Methods and Supplementary Section 5 for a full description of the detection and processing of data for the SPR reflectance). With the current device thickness *t*_glass_ = 1.4 mm, the system can detect shifts up to Δθ_SPR_ = 4.8° (Δ*x*_SPR_ ≈ 1600 μm), which corresponds to a bulk refractive index of *n*_s_ = 1.379. In the planar geometry of our device, this dynamic range can be easily varied by changing the thickness *t*_glass_ according to eq 2 (see Figures S11, S12 and Supplementary Section 6 for details on the sensitivity scaling in the planar geometry).

**Figure 4:**
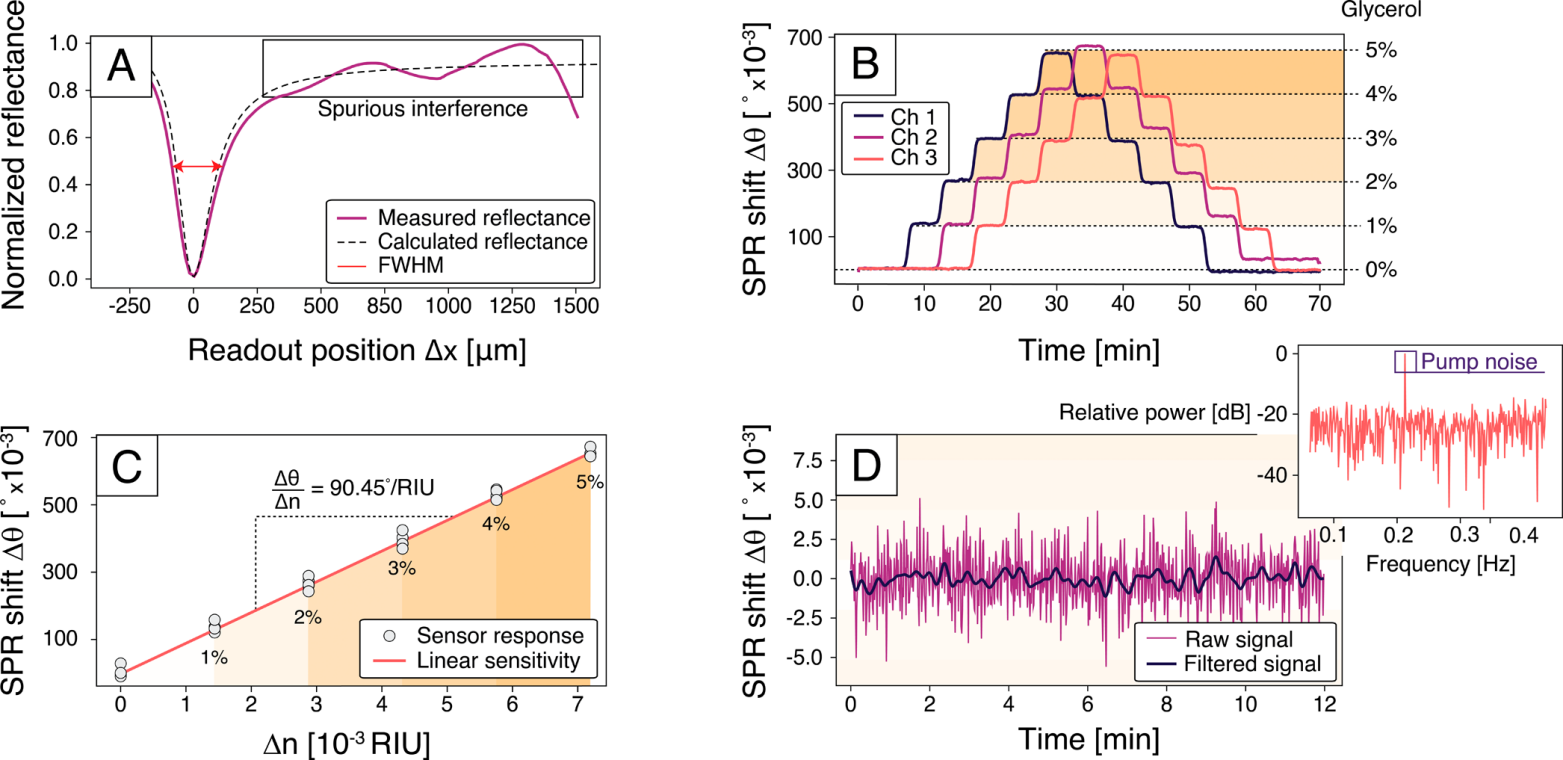
Sensor readout, bulk sensitivity, and resolution. (A) Integrated outcoupled intensity versus readout position Δ*x* for one sensor channel filled with pure water (*n*_s_ = 1.333), compared with the corresponding theoretically calculated intensity distribution. (B) SPR angle shift Δθ_SPR_ recorded over time for three individually addressable channels during continuous flow-through experiments using glycerol solutions. The glycerol concentration was increased sequentially in increments of 1%. (C) Relationship between SPR angle shift (Δθ_SPR_) versus refractive index increment (Δ*n*) relative to pure water, extracted from the data in B. The linear fit indicates a clear linear dependence and enables quantification of bulk sensitivity. (D) Drift analysis of SPR angle (Δθ_SPR_) during continuous flow of pure water at a rate of 30 μL/min. The inset shows the corresponding power spectrum of the signal, revealing a prominent peak at ≈ 0.2 Hz attributed to the peristaltic pump. After suppression of this peak through low-pass filtering, the noise level corresponds to a bulk refractive index resolution of *R* = 4.9 × 10^–6^ RIU.

Figure 4B shows measurements in which glycerol in water solutions (0–5% glycerol in 1% increments) was flowed consecutively into the three microfluidic channels of the device. The recorded SPR responses show clear steps, matching the different perfused solutions with excellent baseline stability. The staggered injections show that there is no crosstalk between adjacent channels in the presence of both a higher and lower concentration analyte. Linear regression of the response (Figure 4C) yields an experimental bulk sensitivity of $S_{\mathrm{bulk}}=\frac{\Delta \theta_{\mathrm{SPR}}}{\Delta n_{s}}$ = 90.45°/RIU, which is close to the theoretically predicted value from eq 1 (*S*_bulk_ = 94.3°/RIU at *n*_s_ = 1.333).

The bulk refractive index resolution, *R*, of our device was estimated by measuring the standard deviation σ_θ_ in Δθ_SPR_(*t*) during the continuous flow of pure water through a sensing channel. The resolution can then be estimated as

$$R=\frac{\Delta n_{s}}{\Delta \theta_{\mathrm{SPR}}}\sigma_{\theta }=\frac{\sigma_{\theta }}{S_{\mathrm{bulk}}}$$ 3

Using the experimental *S*_bulk_ above, the 12 min time trace, shown in Figure 4D yields σ_θ_ = 1.6 × 10^–3^ °, which corresponds to *R* = 1.80 × 10^–5^ RIU. However, the frequency spectrum of the SPR response (Figure 4D inset) reveals a distinct peak at ≈ 0.2 Hz, corresponding to the rotation frequency of the peristaltic pump. When the pump is turned off, the noise is significantly reduced, and a resolution of *R* = 4.6 × 10^–7^ RIU is achieved (Figures S13, S14 and Supplementary Section 7). This suggests the possibility to improve the resolution of the device by employing an alternative pumping or microfluidic scheme that can provide a steadier flow, such as a syringe or a gravity-driven pump.^28^ In the subsequent experiments, the strong peak from the pump was filtered out by a long-pass filter with a cutoff at 0.1 Hz. This approach suppresses the pump noise while still preserving the slower kinetics of typical biochemical interactions. The filtered time trace is presented in Figure 4D demonstrating an improved standard deviation, σ_θ_ = 4.43 × 10^–4^ °, and resolution, *R* = 4.9 × 10^–6^ RIU.

### Surface Sensitivity Characterization Using Protein Multilayers

To assess the device’s capability for resolving thin biomolecular layers, we monitored the stepwise assembly of protein monolayers using electrostatically adsorbed bovine serum albumin (BSA). This layer-by-layer approach involves alternating exposure to positively charged BSA and negatively charged dextran sulfate (DS), which reverses the surface charge to enable subsequent electrostatic adsorption of BSA^29^ (see Methods for assay details). Each BSA layer is expected to form a homogeneous film approximately 5 nm thick, given by the molecular diameter of BSA.

Figure 5A illustrates the formation of two BSA monolayers. After adsorption of the first layer and rinsing with buffer, the surface is recharged with DS, enabling the adsorption of the next BSA layer. The gradual saturation in the sensor response Δθ_SPR_(*t*), observed during each deposition step, is consistent with electrostatic repulsion between surface-bound proteins, leading to termination at monolayer coverage. Figure 5B shows the cumulative SPR angle shift during the formation of eight successive BSA layers. Interestingly, the incremental SPR angle shift per layer increases with the total number of deposited layers, an initially counterintuitive observation given the evanescent decay of the surface plasmon field. Since the penetration depth of the SPR field into the dielectric medium is limited, one might expect reduced sensitivity for material located farther from the gold interface. However, this nonlinear trend arises from the high refractive index contrast between the protein *n*_BSA_ = 1.44, and the surrounding buffer *n*_buffer_ = 1.34, coupled with the inherently nonlinear dependence of the SPR angle on the local refractive index profile (eq 1). As shown in Figure 5C, our experimental results are in excellent agreement with electrodynamic simulations performed in COMSOL Multiphysics (Figure S7), assuming a uniform BSA layer thickness of 5 nm. The initial BSA layer directly adsorbed onto the gold surface is not included in the quantitative analysis, as its binding mechanism differs from the electrostatic interactions governing the subsequent BSA–BSA layers. While the first layer likely adheres via a combination of hydrophobic and van der Waals interactions, the additional BSA layers form through electrostatic attraction, which supports more consistent and interpretable modeling. The simulations reproduce the observed nonlinear increase in SPR angle with layer number and validate the device’s ability to quantitatively resolve nanoscale variations in surface-bound material (see Supplementary Section 4 for simulation details).

**Figure 5:**
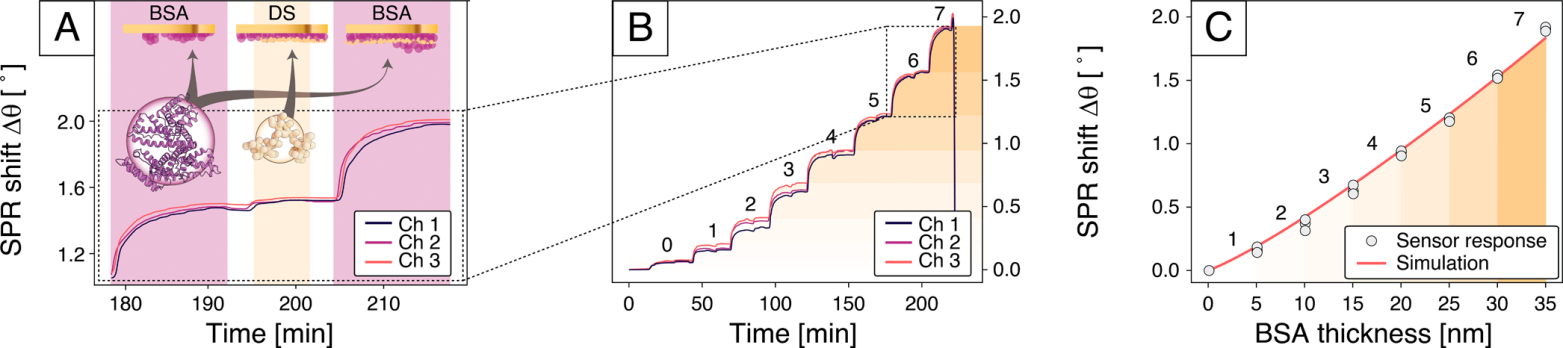
Detection of thin biomolecular layers. (A) Expanded view showing the formation of the sixth and seventh bovine serum albumin (BSA) layers (highlighted in purple). Each protein adsorption step is followed by a rinsing step with buffer solution, surface activation by dextran sulfate (DS, beige region), and a final rinse, which stabilizes and prepares the surface for subsequent protein deposition. (B) SPR angle shift recorded in real time during the sequential formation of eight BSA monolayers across three independent sensor channels (black, purple, and orange curves). (C) Comparison of experimentally measured and simulated SPR shifts as a function of the cumulative thickness of the BSA multilayers. The simulation assumes that each BSA layer has a thickness of *t*_BSA_ = 5 nm and an effective refractive index of *n*_BSA_ = 1.44. The close agreement between measurement and simulation confirms the accuracy and sensitivity of the sensor in quantifying protein layers.

### Biomolecular Analysis and Limit of Detection

As an application demonstration, we turned to detection of a specific biomolecular target, micro-RNA 122 (miR-122), a known circulating biomarker for drug-induced liver injury and other hepatic disorders.^30^ The detection assay, adapted from ref 29 and described in detail in the Methods section, is performed in three sequential steps. First, the gold sensor surface is functionalized with 5*′*-thiolated single-stranded DNA probes complementary to miR-122, enabling covalent attachment to the gold film. The remaining gold surface is blocked with mercaptohexanol (MCH) to prevent nonspecific binding. After flushing all three sensing channels with buffer to establish a baseline, two channels are exposed to buffer solutions containing varying concentrations of miR-122, allowing specific hybridization and formation of RNA–DNA duplexes. The third channel served as a negative control and was exposed to a miRNA-free buffer. In the final step, all channels were perfused with antibodies selective for RNA-DNA hybrid duplexes. These antibodies, due to their substantially higher molecular weight compared to miR-122, significantly enhanced the surface plasmon resonance (SPR) signal, thereby improving detection sensitivity at low analyte concentrations. Figure 6 summarizes the assay results (see Figure S15 and Supplementary Section 8 for the assay details and full traces). Measurements were performed using miR-122 concentrations ranging from 0.01 to 5 nM in logarithmic steps. Each concentration was tested in duplicate or triplicate for lower concentrations, using independent sensor chips to confirm reproducibility. For the investigated concentration range, the sensor response Δθ_SPR_(*t*) increases linearly with time (Figure 6A), which implies that the interaction is diffusion-limited.^29^ To quantify the detection limit, responses from the reference channel were subtracted for both the hybridization and antibody binding steps (Figure S6). The limit of detection (LoD) for direct hybridization-based sensing was estimated from the sensor response at *t* = 15 min, i.e., at the end of the incubation period with the miRNA sample. The calibration curve was calculated using linear regression, yielding a LoD ≈ 0.1 nM (Figure 6B), which would be the sensor response corresponding to the standard deviation of the baseline signal (Figure 4D). Similarly, the detection limit using antibody amplification was calculated at the plateau of the response, at *t* = 32 min in Figure 6A, which by comparison to standrad devication gives a LoD ≈ 0.02 nM with sensitivity on par with or exceeding existing SPR-based miRNA detection platforms.^31^

**Figure 6:**
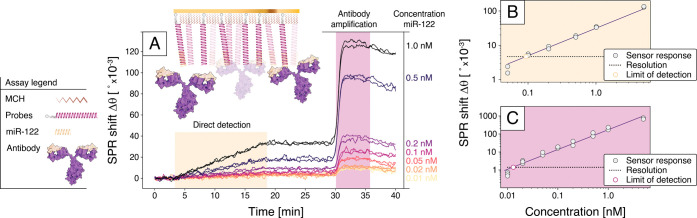
Specific detection of microRNA-122 (miR-122) using direct and antibody-amplified SPR biosensing. (A) Time trace of the miR-122 binding assay, accompanied by a schematic of the detection mechanism. DNA probes complementary to miR-122 were immobilized on the gold surface via hexanethiol linkers. The immobilization occured prior to the presented time trace. The first 3 min established a baseline in Tris_Mg_ buffer, followed by introduction of the target miR-122 and formation of miRNA–DNA duplexes over 15 min. After a subsequent buffer rinse, a monoclonal antibody specific to the RNA–DNA duplexes was introduced to amplify the SPR signal. (B) SPR sensor response at *t* = 18 min (direct detection without amplification) plotted as a function of miR-122 concentration. Data points are presented on a log–log scale along with a linear fit, demonstrating the sensor’s concentration-dependent response. (C) Antibody-amplified SPR sensor response at *t* = 32 min as a function of miR-122 concentration in a log–log plot together with a linear regression fit. Horizontal dashed lines in panels (B) and (C) indicate the SPR sensor’s resolution limit, derived from Figure 4D, resulting in miR-122 limits of detection (LoD) of 0.1 nM for direct detection (B) and 0.02 nM for antibody amplification. All data were corrected for signal drift and nonspecific interactions by subtracting the response from a reference channel in which a blank sample was injected. Each concentration was measured in 2–3 replicates across different SPR chips.

## Discussion

Surface plasmon resonance (SPR) has long been recognized for its label-free detection capabilities and high sensitivity, yet its widespread use in portable, low-cost biosensing systems has remained limited due to the size, complexity, and cost of conventional optical setups. Our work addresses these challenges through the development of a miniaturized, flat SPR biosensor that integrates metasurface optics, semiconductor laser arrays, and microfluidics on a single platform. This device exemplifies a growing trend in the field of metaoptics: the shift from isolated component innovations to fully integrated, system-level solutions.

In contrast to the great majority of conventional angle-resolved SPR systems that rely on bulky prism-based setups and tightly focused beams,^5,8,32^ our design employs a divergent laser beam that illuminates the sensor surface along a line.^22^ This enables spatial encoding of the SPR excitation angle where the resonance condition shifts laterally with changes in the refractive index. As a result, the SPR signal can be extracted directly from a single camera image, eliminating the need for mechanical scanning or angular tuning. This approach simplifies the optical design and facilitates fast, multiplexed readout using compact CMOS or CCD sensors. Although this spatial encoding introduces some nonlinearity for large refractive index shifts, these effects are negligible for the small refractive index changes typical of biomolecular interactions. Instead, the primary performance limitation in our current setup is the signal-to-noise ratio, which is limited mainly by fluidic instabilities and internal reflections. These factors contribute to a refractive index resolution approximately an order of magnitude lower than what is possible to achieve with state-of-the-art equipment,^33,34^ see Table S1 in Supplementary Section 9 for a comparison with state-of-the-art and compact SPR instrumentation. Moreover, the lasing wavelength of the VCSEL is dependent on the ambient temperature and bias current. Thermal stabilization from a heat sink or a Peltier cooler might therefore be needed to achieve similar performance in a metalaser device used outside of the laboratory setting. Nonetheless, the significant benefits in terms of miniaturization, integration, and manufacturing scalability outweigh this limitation, particularly in the context of portable or resource-limited diagnostic settings.^35^ Our proof-of-concept implementation relied on an inverted microscope for readout, but the optical layout is fully compatible with compact, on-chip imaging sensors. Together with a simplified microfluidic interface, this would enable the development of a fully portable, battery-powered SPR system.

In terms of assay performance, surface plasmon resonance is a mature technology, and a miniaturized device can leverage the wide range of detection assays and surface chemistries already established for real-world applications. In this work, we employed standard thiolated DNA probes with MCH blocking. The sensing surface can be regenerated following the protocol described in ref 29, enabling at least five reuse cycles of the same DNA probes. The intrinsic specificity of the miRNA-DNA probe interaction minimizes cross-hybridization with noncomplementary sequences. However, the surface remains only moderately resistant to nonspecific protein adsorption, including antibody binding. These effects are largely mitigated by referencing against a parallel blank channel, which corrects for the contribution of nonspecific interactions. To further improve sensitivity, quantitative accuracy, and long-term reusability, future implementations could integrate advanced antifouling strategies, such as zwitterionic polymer brushes and ultralow-fouling hydrogels, that are now being developed for SPR applications.^36^

In addition, the system architecture is inherently scalable, while the current implementation features three independently addressable sensing channels, both the metalaser array and the microfluidic layout can easily be expanded to support high-density multiplexing. This capability is increasingly important for new biosensing applications that demand higher throughput.^37^ Moreover, future iterations could extend the system’s functionality toward SPR imaging or microscopy,^38^ enabling spatially resolved, parallel analysis of multiple biomolecular interactions across the sensing surface.^39^ Finally, although our prototype operates at a fixed wavelength of 984 nm, set by the GaAs-based VCSELs, laser arrays at alternative wavelengths are commercially available.^40^ This would open the door to miniaturized multiwavelength or spectroscopic SPR sensing, which could further improve molecular specificity, enable broader target compatibility, and enhance quantitative analysis.^41^

## Summary and Conclusions

We have presented a proof-of-concept miniaturized and flat SPR biosensor that leverages VCSELs monolithically integrated with optical metasurfaces to enable on-chip surface plasmon excitation. Each metasurface reshapes the vertically emitted Gaussian beam from the laser into a fan-like illumination profile, aligned with the SPR resonance condition at the gold–water interface. This architecture eliminates the need for bulk optics or mechanical scanning and enables direct spatial readout with a camera. The device is composed of two modular units: a reusable optical module for illumination and signal outcoupling, and a microfluidic module for analyte delivery. This configuration supports ease of use, reusability, and scalable manufacturing. We characterized the system’s performance by measuring its bulk refractive index resolution, achieving a sensitivity of *R* = 4.9 × 10^–6^ RIU, resolving nanoscale protein multilayers, and detecting the liver disease-associated biomarker microRNA-122 (miR-122). The platform achieved detection limits of 0.1 nM via direct hybridization and 0.02 nM using antibody-based signal amplification. These values are competitive with existing compact SPR systems and are well within the clinically relevant range for miRNA biomarkers. To our knowledge, this is the first demonstration of a metalaser SPR platform that supports multiplexed, camera-based readout in a compact and modular format. While current limitations in signal-to-noise ratio are primarily due to fluidic noise and internal reflections, these can be addressed through further engineering of both metasurfaces and fluidics. With continued development, this platform could evolve into a fully self-contained, battery-powered biosensor suitable for field deployment, point-of-care diagnostics, and even home-based health monitoring. Its compact footprint, scalable architecture, and application-ready sensitivity make it a promising step toward next-generation SPR sensing.

## Methods

### Detection of the SPR Shift

The reflected instensity distribution was captured by magnifying the outcoupling region with a 4× objective (Nikon) and imaging with a CCD camera (Hamamatsu-C11440). For one measurement point in the presented sensor response, 10 images were taken with 30 ms exposure time over the span of 1 s. All of the 10 images are individually processed to find the SPR position in the reflected intensity distribution, and finally, the reported position of the SPR for each second was estimated by averaging the position from all 10 images. The fabricated metalasers are individually biased with 1 mA from a sourcemeter (Keithley-2400) during the measurement.

### BSA Monolayers for Surface Sensitivity

Surface sensitivity was assessed by stepwise assembly of bovine serum albumin (BSA) monolayers using electrostatic layer-by-layer deposition. BSA (0.5 mg/mL) and dextran sulfate (DS, 1 mg/mL) were dissolved in 10 mM citrate buffer (CB, pH 4.0) and flowed sequentially through the microfluidic channels at 30 μL/min and 25 °C. Each adsorption step lasted 10 min, followed by a 5 min buffer rinse. BSA, carrying a net positive charge at pH 4, adsorbed directly onto the gold surface.

Subsequent DS exposure reversed the surface charge, enabling the next BSA layer to bind. This cycle was repeated to form up to eight layers. Real-time SPR measurements were used to track layer growth, and the cumulative signal was compared to a multilayer model assuming 5 nm thickness and refractive index 1.44 per BSA layer.

### miR-122 Assay

The gold surface was functionalized by 5*′*-thiolated DNA probes at 4 μM in phosphate-buffered saline (PBS; Sigma-Aldrich, pH 7.4). The sequence of ssDNA probes was: HS-(CH_2_)_6_-5*′*-CTA GGA TTA GCT ATT TAC TGA GAG AAC GAA GTA TCA TAG TAT AGA GT*C AAA CAC CAT TGT CAC ACT CCA* - 3*′*, where the thiol group (HS) enables immobilization on the gold surface, and the italicized region denotes the sequence complementary to miR-122. The sequence of the synthetic miR-122 was: 5′-r­(UGG AGU GUG ACA AUG GUG UUU G)-3′. All oligonucleotides were obtained from IDT at HPLC purification grade. Probes were immobilized at 6 μL/min for 20 min in all three sensing microfluidic channels pumped by a peristaltic pump (Ismatec).

The surface was then passivated with 1 mM 6-mercapto-1-hexanol (MCH; Sigma-Aldrich) in PBS for 15 min. Synthetic miR-122 was diluted in Tris_Mg_ buffer (10 mM Tris, 30 mM MgCl_2_, pH 7.4) to concentrations ranging from 0.01 to 10 nM. The solution was flowed through two sensing channels at 30 μL/min for 15 min to enable hybridization with surface-bound probes.

All steps were carried out at room temperature (22–24 °C). After hybridization, Tris_Mg_ buffer was flushed through all channels for 10 min. A mouse monoclonal anti-DNA-RNA duplex antibody (Clone S9.6, Millipore, Cat. #MABE1095, 5 μg/mL in Tris_Mg_) was then introduced at 30 μL/min for 5 min. Specific responses were obtained by subtracting the reference channel signal and averaging the postincubation plateau. Each concentration was tested in 2–3 replicate channels across at least two independently prepared chips, with no surface regeneration between measurements.

## Supplementary Material



## Data Availability

The data supporting the findings of this study are available from the corresponding author upon reasonable request.

## Notes

Images of the full sensor; fabrication processes; simulations; SPR tracking method; noise characterization; description of response calculations; and a table for comparison with other compact SPR instrumentation (PDF)
