# Supplementary material for: Flat Plasmonic Biosensor with an On-Chip Metagrating-Integrated Laser
Source: ACS Sens. 2025 Oct 8;10(10):7670–8. doi: 10.1021/acssensors.5c01997 (PMC12560135; doi:10.1021/acssensors.5c01997)
Supplement: Supplementary file 1 [file se5c01997_si_001.pdf]

Flat Plasmonic Biosensor with an On-Chip Metagrating-Integrated Laser - Supplementary information

Erik Strandberg[1, *], Mindaugas Juodėnas[2, 3], Hana Šípová-Jungová[3], and Mikael Käll[3]

[1]Department of Microtechnology and Nanoscience, Chalmers University of Technology 412 96 Gothenburg
[2]Institute of Materials Science, Kaunas University of Technology 514 23 Kaunas
[3]Department of Physics, Chalmers University of Technology 412 96 Gothenburg
[*]Corresponding author: erik.strandberg@chalmers.se
Contributing authors: mindaugas.juodenas@ktu.lt; hana.jungova@chalmers.se; mikael.kall@chalmers.se

Images of full sensor

Images of the full sensor placed on the sample stage of an inverted microscope.

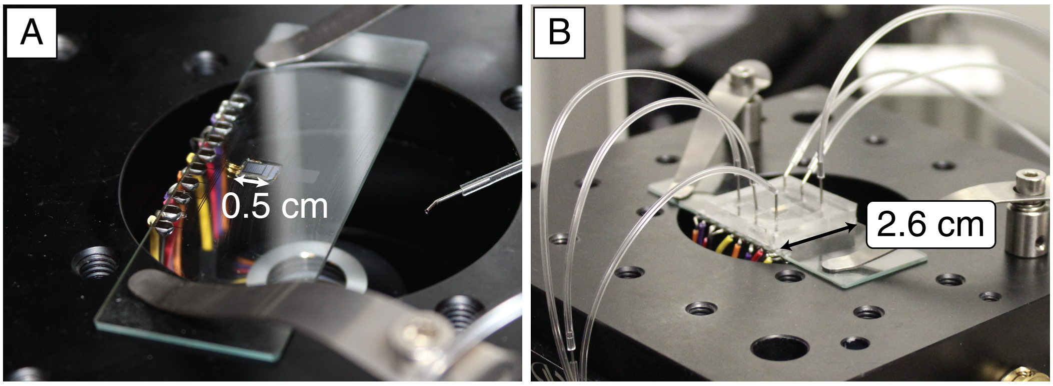

Figure S1: Camera images of the full sensor module. **A)** The bare sensor with the metalaser-array chip bonded to a glass slide with the Au lines for outcoupling of light. The small 0.5x0.5 cm chip contains 81 metalasers that can be individually addressed for surface plasmon excitation. **B)** The sensor module on the sample stage of an inverted microscope, with the interfaced PDMS microfluidics channels and tubing for the peristaltic pump.

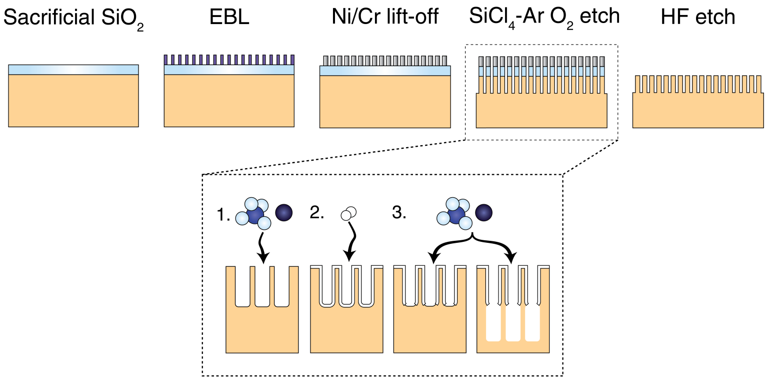

Figure S2: Process flow for the monolithically integrated metagrating from left to right. First 25 nm of sacrificial SiO_2 is sputtered on the GaAs surface. The metagrating pattern is exposed in the positive electron-beam resist, ArP-6200, and a metal mask of nickel-chromium is created via lift-off. The metagrating is etched into the GaAs with a switched $SiCl_4$ and O_2 process, and finally the metal is removed by wet etching in HF.

1 Metalaser fabrication

The vertical-cavity surface-emitting laser (VCSEL) cavity is designed using the effective index method [1]. Briefly, the VCSEL epitaxial structure features three strained InGaAs quantum wells sandwiched between two distributed Bragg reflectors (DBRs). The top DBR has one AlGaAs layer with an aluminum content of 98.5%, which is selectively oxidized to form an oxide aperture. The target aperture diameter is 2 μm to achieve single-mode lasing. For metagrating fabrication, a SiO_2 layer was sputtered on the substrate side to serve as a sacrificial layer for mask removal. Optimized patterns for the metagrating were exposed using electron beam lithography. After development, the Cr-Ni metal mask was evaporated. The patterns were etched into SiO_2 in CF_4/O_2 plasma, followed by a GaAs etch using the switching process alternating between the $SiCl_4$ and O_2 plasma processing steps, as seen in **Fig. S2** [2].

2 Metasurface optimization and characterization

The periodicity of the metagrating is larger than the wavelength in the glass substrate, which allows for the diffraction of light into the zeroth and first diffraction orders, $+1$, 0 and -1, as seen in **Fig. S3A** [3]. Each unit cell contains several scatterers that can be optimized to give destructive interference in the unwanted diffraction orders, 0 and -1, and constructive interference in the single desired diffraction order, $+1$. The metagrating is designed using the wave optics module in the commercial electrodynamic simulation tool COMSOL Multiphysics. The metagrating is simulated in 1D using periodic boundary conditions and plane wave illumination, and the resulting field is presented in **Fig. S3B**. The position and height of the ridges in the simulation domain are optimized to create the interferences needed to maximize the relative transmission efficiency, η_{rel}, for diffraction order T_{+1}:

$$\eta_{rel} = \frac{T_{+1}}{T_{+1} + T_0 + T_{-1}},$$

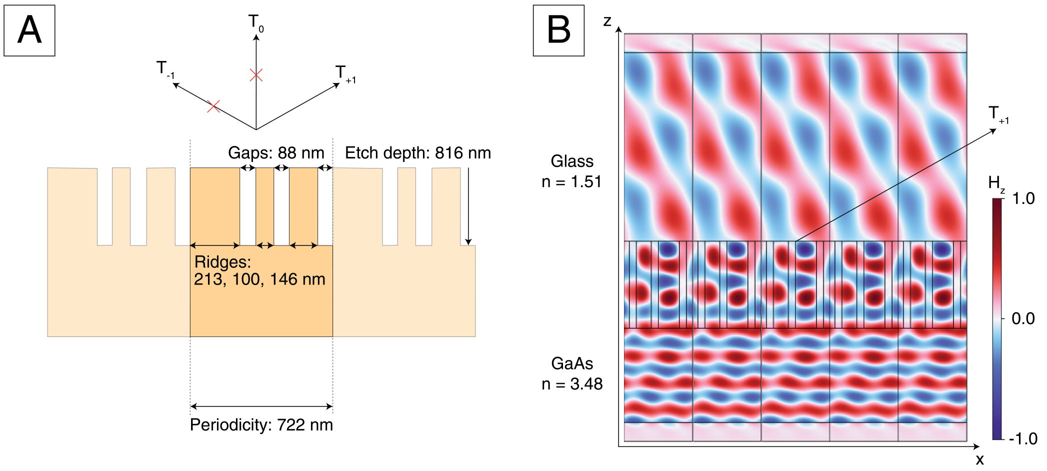

Figure S3: **A)** Presents the optimized dimensions of the fabricated metagrating. The equal gaps in the grating ensure that the entire grating is etched to the same depth, which circumvents aspect-ratio dependent etching, associated with monolithic integration. The height and the size of the ridges are optimized to create destructive interference for T_0 and T_{-1} diffraction, channeling almost all light into T_{+1}. **B)** Shows the orthogonal component of the magnetic field, H_z, from the COMSOL simulation for the optimized metagrating. The metagrating is illuminated by a plane wave from the GaAs-side and clear diffracted wavefront can be seen propagating in the first diffraction order, T_{+1}, with the angle, $\theta_{+1} = 64.49°$, into the glass environment. The relative transmission efficiency is $\eta_{\mathrm{rel}} = 0.76$.

where $T_{+1},\ T_0,\ T_{-1}$ is the transmission into each diffraction order. The optimization is made under the constraint that all the gaps should have the same width. This constraint ensures that all the ridges will be etched to exactly the same depth, which circumvents any aspect-ratio-dependent etching. The angle of the diffracted beam is determined by the grating equation

$$\sin\theta_m = \frac{m\lambda_0}{nP}$$

where m is an integer for a certain diffraction order, λ_0 is the wavelength in free space, n is the refractive index of the transmitted environment, and P is the periodicity of the grating. The periodicity sets the deflection angle along the x-axis. By curving the optimized structures into concentric circles, we transform a linear phase gradient into a conical one, which is the same as an axicon's phase profile. The offset for the conical phase profile of the axicon from the aperture of the VCSEL, x_0, will set the curvature radius of the grating lines. The imparted phase profile of the offset axicon will be linear along the x-axis, which corresponds to deflection, and hyperbolic along the y-axis, hence producing a focusing effect for the beam along the latter. A larger offset, x_0, will give a larger curvature, which in turn leads to a stronger lensing, or a shorter focal length. The focal length corresponding to a certain x_0 is found by comparing the phase profile produced by an axicon and the hyperbolic phase profile of a lens. The conical phase profile that can be offset by x_0 from the VCSEL aperture is

$$\phi_{\mathrm{axicon}}(x,y) = -\frac{2\pi n_{\mathrm{out}}}{\lambda_0}\mathrm{NA}\sqrt{(x-x_0)^2+y^2}$$

where n_{out} is the refractive index of the medium in which the beam is transmitted and λ_0 is the wavelength in free space. A certain offset, x_0, will make the difference between the hyperbolic phase

distribution of the offset axicon and the hyperbolic phase from the lens with a certain focal length, f, approach zero along the y-axis as

$$\begin{aligned}\Delta\phi(0,y) &= \phi_{\text{lens}}(0,y) - \phi_{\text{axicon}}(0,y) \to 0 \\ &= n_{\text{out}}\text{NA}\sqrt{x_0^2+y^2} - n_{\text{f}}\left(\sqrt{y^2-f^2}-f\right) \to 0,\end{aligned} \tag{1}$$

where n_{f} is the refractive index of the medium for which the focal length is set. The equation above can be solved to find the offset x_0 to achieve a certain focal length, f, for the hyperbolic phase profile of the offset-axicon metagrating. In our design, the focal length is set to $f = 600\ \mu$m which counteracts the inherent divergence of the beam from the VCSEL and collimates the beam along the y-axis.

To characterize the performance of the metalaser, it was bonded to a 0.4 mm thick glass slide using a refractive index matched optical adhesive (Norland NOA60). The Fourier plane of the produced beam is imaged by a lens focused on the back focal plane of a 100x, NA= 1.49, immersion objective, as seen in **Fig. S4A**. The measured relative transmission efficiency of the lasers was determined by setting a threshold for the background, from the surrounding room and the spontaneous emission from the VCSEL, and integrating the counts for each diffraction order in the Fourier plane image, Figure **Fig. S4B**.

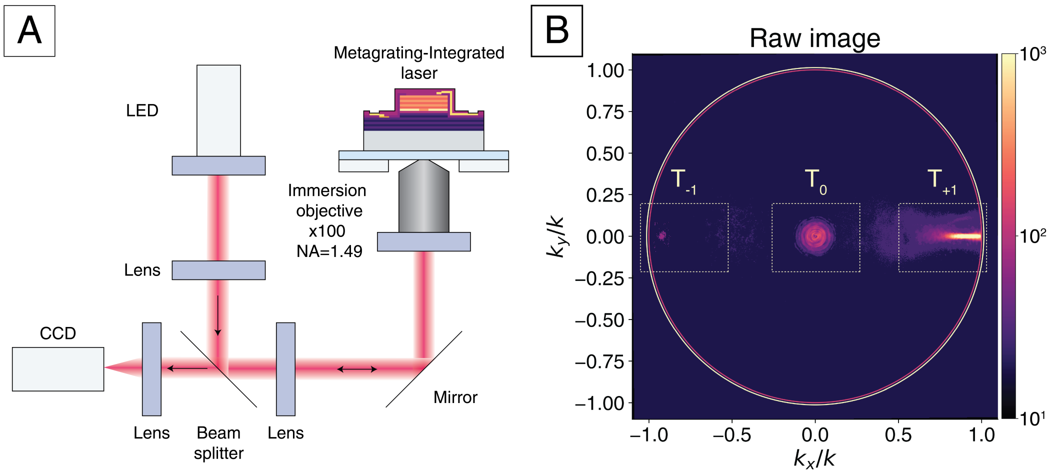

Figure S4: A) The inverted microscope stage used for the metagrating-integrated VCSEL characterization, with the laser bonded to a thin glass slide to be able to image the beam with a high numerical aperture, NA= 1.49, immersion objective. **B)** The raw Fourier plane image captured by the CCD camera on the inverted microscope stage in logarithmic scale. The marked line in the image corresponds to the numerical aperture for the objective. The efficiency is calculated by setting a threshold for the background intensity from the environment and the spontaneous emission of the VCSEL, and then integrating the counts in the three different diffraction orders, integration regions are marked with the yellow squares.

3 SPR chip fabrication

The fabrication process for the SPR slides is presented in **Fig. S5A**. The sensor slides of approximately 0.4 mm thickness are thoroughly cleaned by solvent cleaning and a strong oxygen plasma before evaporating three stripes of 2/50 nm Ti/Au with dimensions 600x6000 μm for surface plasmon polaritons (SPP) excitation. The Ti is an adhesion layer for the Au film to the glass surface; the Ti does not have the same plasmonic properties as Au and therefore reduces the intensity dip from the SPR in the reflected intensity spectrum, but 2 nm worked as a good trade-off between managing to detect a significant intensity dip and the Au film not peeling during the miRNA assay.

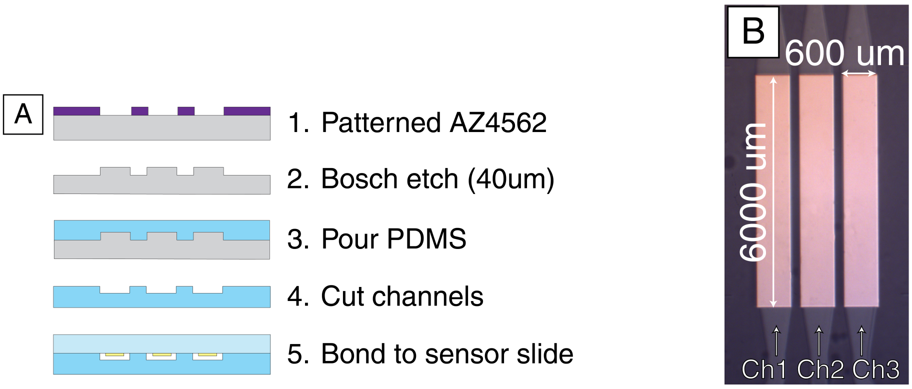

Figure S5: A) Fabrication process for the SPR chips. **B)** Microscope image of the gold strips for SPP excitation with the PDMS channels bonded.

For the polydimethylsiloxane (PDMS) microfluidics fabrication, a master mold was fabricated in silicon. The mold is patterned with AZ4562 and etched by a standard deep reactive ion etching Bosch process to a height of 40 μm. The PDMS is mixed with a curing agent in a ratio of 10:1, poured into the master mold, and degassed. When the PDMS is bubble-free, it is cured on a hot plate at 100° for 60 min. After which, holes in the PDMS were cut for tubing, and the PDMS side was reactivated by an oxygen plasma for 30 s. The patterned glass slide with the sensor stripes was bonded by gently pressing and baking the full sensor slide on a hot plate at 100° for 5 min. The Au stripes were patterned with some spacing between each other, leaving some space for the PDMS to bond to a bare glass surface and form a tight seal for the channel. Microscope image of the SPR chip with the gold sensing strips and the bonded PDMS microfluidics is presented in **Fig. S5B**.

4 SPR reflectance simulation

4.1 Reflectance for a homogeneous medium

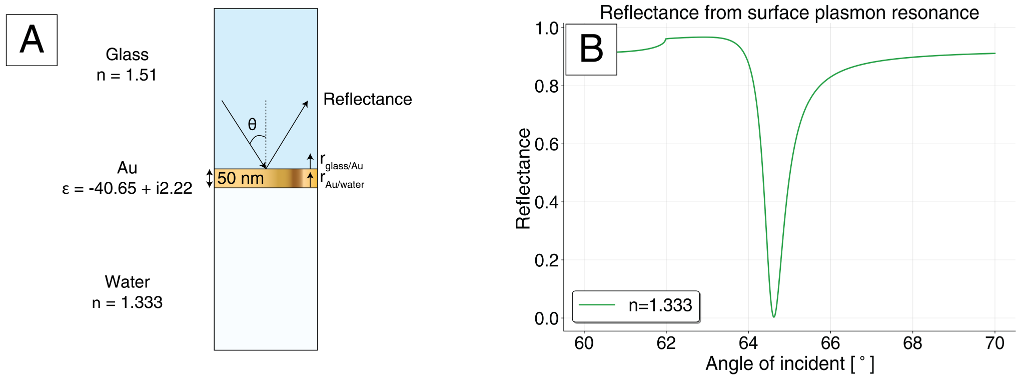

Figure S6: A) The multilayer structure with the gold film, glass substrate and sensing channel which the reflectance is calculated for. **B)** Calculated reflectance from Fresnel multilayer theory used to predict the angle of the surface plasmon excitation.

The gold film reflectance spectrum is calculated for the Kretschmann geometry using Fresnel multilayer theory [4]. Briefly, the reflectance is calculated as

$$R = |r|^2, \quad r = \frac{r_{\text{glass/Au}} + r_{\text{Au/s}} e^{i2k_{\text{Au}}t_{\text{Au}}}}{1 + r_{\text{glass/Au}} r_{\text{Au/s}} e^{i2k_{\text{Au}}t_{\text{Au}}}},$$

where t_{Au} is the thickness of the gold film, $r_{\text{glass/Au}}$ is the field reflection coefficient for the glass-metal interface

$$r_{\text{glass/Au}} = \frac{\varepsilon_{\text{Au}} k_{\text{glass,z}} - \varepsilon_{\text{glass}} k_{\text{Au,z}}}{\varepsilon_{\text{Au}} k_{\text{glass,z}} + \varepsilon_{\text{glass}} k_{\text{Au,z}}},$$

and $r_{\text{Au/s}}$ is the field reflection coefficient for the metal interface to the sensing channel

$$r_{\text{Au/s}} = \frac{\varepsilon_{\text{s}} k_{\text{Au,z}} - \varepsilon_{\text{Au}} k_{\text{s,z}}}{\varepsilon_{\text{s}} k_{\text{Au,z}} + \varepsilon_{\text{Au}} k_{\text{s,z}}}.$$

The wavenumber in each medium is calculated as:

$$k_{\text{m,z}} = \sqrt{\varepsilon_{\text{medium}} \left(\frac{2\pi}{\lambda}\right)^2 - k_{\text{x}}^2},$$

where the k_x is the in-plane wavenumber of the incident beam

$$k_{\text{x}} = \left(\frac{2\pi}{\lambda_0}\right) n_{\text{glass}} \sin(\theta).$$

To estimate the angle for the excitation of surface plasmons, we used the refractive index of the glass substrate, $n_{\text{glass}} = 1.51$, that the metalaser array is bonded to, the complex permittivity of the gold film, $\varepsilon_{\text{Au}} = -40.65 + i2.22$, at $\lambda_0 = 984$ nm [5], and finally, pure water is assumed to have the refractive index, $n_s = 1.333$. The multilayer structure and the calculated reflectance as a function of the incident angle are presented in **Fig. S6**.

4.2 Reflectance from thin layer of bovine serum albumin

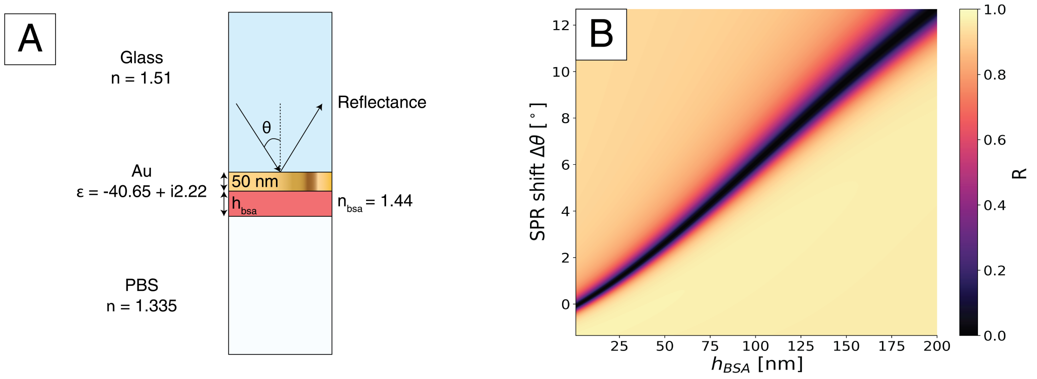

Figure S7: A) Structure simulated in COMSOL with the BSA-DS layers approximated as a single homogeneous medium with an effective refractive index, $n_{\text{bsa}} = 1.44$. **B)** The reflectance as a function of the incident angle of the illuminating plane wave and the height of the bound BSA layers, h_{BSA}. The SPR resonance angle is presented from the initial angle for the reflectance dip without any BSA in the PBS buffer with refractive index $n_{\text{PBS}} = 1.335$. The response has an initial non-linear slope that matches the measured response in **Fig. 5**. The sensitivity can be seen decreasing as the BSA layer approaches the length of the evanescent field.

As explained in the main text, the performance of the device in terms of thin layer sensitivity was tested by an experiment where alternating layers of bovine serum albumin (BSA) and dextran sulfate (DS) were electrostatically bound to the Au surface. The result is presented in **Fig. 5A** in the manuscript. The increase in sensitivity can be predicted from 1D electrodynamic simulations in COMSOL. In the simulations, a BSA-DS layer is approximated as a uniform medium with an effective refractive index n_{BSA}. The simulations are performed by capturing the resulting reflectance while illuminating a gold film, with permittivity $\epsilon_{\text{Au}} = -40.650 + i2.225$ and thickness 50 nm, by a plane wave, $\lambda = 984$ nm, with incident angles ranging from 58.8°-72.8°, the simulated structure is presented in **Fig. S7A**. By running the simulation for several different refractive indices for the phosphate-buffered saline (PBS) buffer and BSA-DS layer, a close match to the measured data was found for $n_{\text{PBS}} = 1.335$ and $n_{\text{BSA}} = 1.44$, the simulated reflectance is presented in **Fig. S7B**. The slight increase in refractive index in the salty buffer, as compared to pure water, is attributed to the presence of more ions in the solution. The refractive index of BSA has been reported to be as high as 1.602 in crystallized form [6].

5 Detection and processing of data for the measurement

The outcoupled intensity distribution is imaged by an inverted microscope with a 4x objective, as seen in **Fig. S8**. The outcoupling gold lines are spaced by 15 μm which gives an angle resolution of approximately 0.2°. The measurement procedure is as follows: images are captured by the camera with an exposure time of 10 ms, as seen in the left image in **Fig. S9**, and stored in the camera buffer. Every 100 ms, 10 captured images are retrieved from the camera and averaged to suppress high-frequency noise. Every 1 s the latest images are averaged again to find the position of the reflectance minimum from the surface plasmon excitation. As seen in **Fig. S9A**, the chips used for the measurements had nine lasers in parallel indicating the potential for immediate scalability; for demonstration purposes, all measurements in the paper were made using only three individual channels.

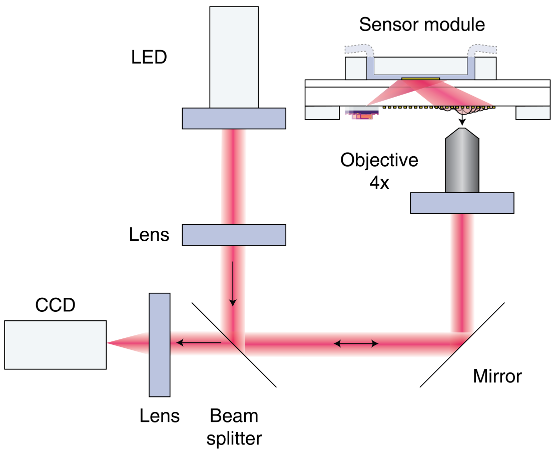

Figure S8: Schematic of the full sensor module on the inverted microscope stage for the SPR measurements. The 40x objective magnifies the outcoupling region on the sensor chip which is then focused down and imaged by an external CCD-camera

The images from each individual channel are averaged over the width of the reflected strip. Since the gold lines discretely sample the reflected intensity, the peaks in the averaged intensity spectrum are picked out for the discrete points where the gold lines are located, as seen in **Fig. S9B**. **Figure S10A** presents the outcoupled intensity distribution from one channel. The resulting spectrum is low-pass filtered to remove some of the inconsistencies in the spectrum, which come from the inhomogeneous scattering of the gold lines and different reflections inside the glass slide. However, the SPR dip is always significant compared to the inconsistent features in the distribution, and the position and the shape of the SPR dip approximately matches the theoretically calculated spectrum with water as the analyte, $n_s = 1.333$, as presented in **Fig. S10B**. Finally, a quadratic fit is performed on the 10 measurement points around the minimum value found in the reflected spectrum, and the minimum value from the quadratic fit is presented as one measurement point.

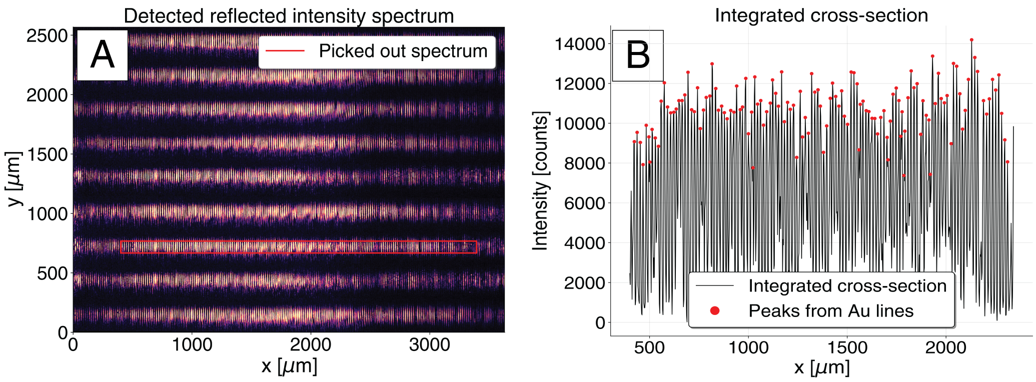

Figure S9: A) A raw image of the outcoupled intensity distribution from nine individually addressable metalasers, indicating the potential for multiplexed measurements. For demonstration purposes, all measurements in the paper were made using only three individual channels. The image is the average of ten images with 10 ms exposures. **B)** Vertically (y-axis) integrated intensity distribution from the metalaser, marked with a red square in **A**. The red dots corresponds to the intensity at each of the outcoupling gold lines.

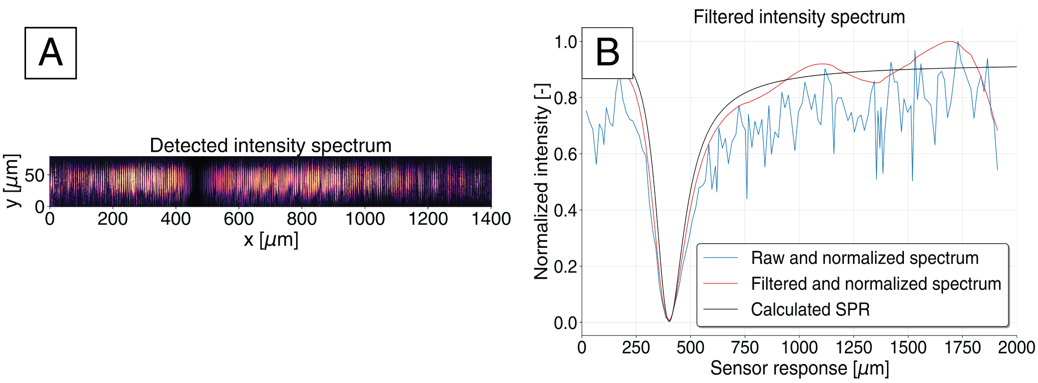

Figure S10: A) A raw image of the outcoupled intensity distribution for one channel filled with water, $n_s = 1.333$. **B)** Vertically (y-axis) integrated intensity distribution with the corresponding filtering for quantification of the SPR position. The position and shape of the SPR dip is in good agreement with the calculated gold film reflectance, though spurious interferences from reflections in the glass slide to which the metalasers are bonded are also evident. Moreover, the SPR dip in the reflectance is clearly the most pronounced feature and it can be easily isolated and tracked. The exact SPR position is obtained through a quadratic fit to 10 camera pixel values around the minimum of the filtered distribution.

6 Sensitivity scaling in the planar geometry

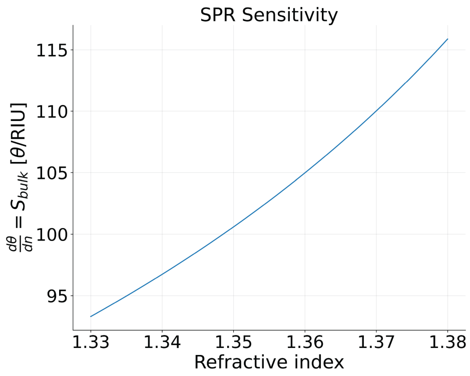

Figure S11: Calculated sensitivity, $\frac{d\theta}{dn}$, for a range of typical sample refractive indices, $n = 1.33$-1.38.

An interesting fact of the SPR sensor in a planar geometry, compared to the conventional prism coupling, in the Kretschmann configuration, is that the sensitivity of the readout for the device scales more strongly with the thickness of the glass substrate, t_{glass}. In **Fig. S11** the calculated bulk sensitivity is plotted as a function of the refractive index in the sensing channel, where the sensitivity is seen to increase with the refractive index. In **Fig. S12** the theoretical spatial position for the readout of the reflectance minimum and the corresponding sensitivity of the spatial shift with respect to refractive index changes is presented in the planar geometry and in the conventional prism design. From **Fig. S12B** it is clear that the refractive index sensitivity of the spatial position readout is more pronounced in the planar geometry. This is due to the projection of the readout, **Eq. 2** in the paper:

$$\theta_{\text{SPR}} = \arctan\left(\frac{x_{\text{SPR}}}{2t_{\text{glass}}}\right)$$

where t_{glass} is the full thickness of the glass slide between the metalaser and the gold film. This effect can be utilized to match the sensitivity range and readout area to a given detector size.

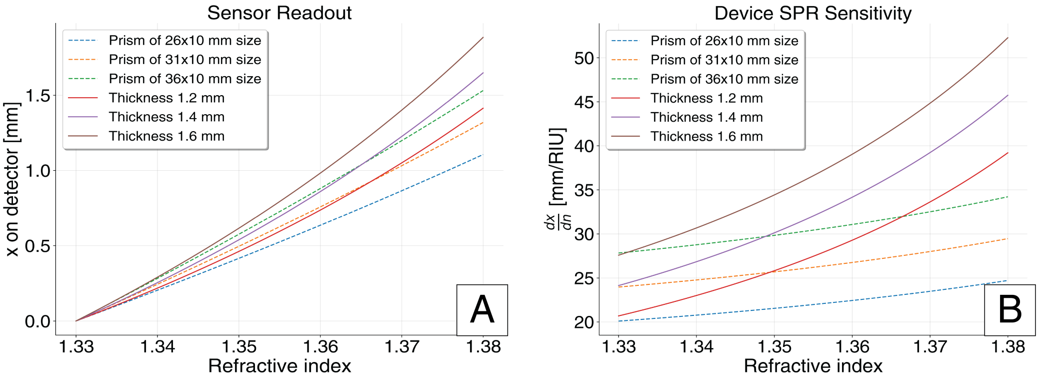

Figure S12: Calculated positions of the reflectance minimum and the corresponding sensitivity for angle-resolved SPR. The spatial position of the SPR dip readout depends on the geometry of the device in the Kretschmann configuration. In the figure, the sensitivity and the position of the SPR readout is theoretically calculated in the planar geometry for three different thicknesses, t_{glass}, and compared to three different sizes of prisms used in conventional SPR. **A)** The spatial position of the SPR dip in the planar geometry exhibits a larger shift with refractive index than in the prism geometry, due to the projection of the readout position, $\theta_{\mathrm{SPR}} = \arctan\left(\frac{x_{\mathrm{SPR}}}{2t_{\mathrm{glass}}}\right)$. **B)** The corresponding readout refractive index sensitivity, $\frac{\mathrm{d}x}{\mathrm{d}n}$, which varies more rapidly with the refractive index in the planar geometry.

7 Sensor sensitivity without the peristaltic pump

As described in the manuscript, the largest noise source in our measurements was the peristaltic pump, **Fig. 4D**. To estimate the best possible resolution of the sensor, milli-Q water was flown into the sensing channel and the pump was turned off. The SPR shift from the still water was tracked for 60 min. In **Fig. S13A**, a 16 min section of the measurement is presented together with the corresponding frequency spectrum in **Fig. S13B**. Without the peristaltic pump, the measurement shows that our device could in principle have a resolution as low as $R = 4.6 \cdot 10^{-7}$ RIU. **Figure S14A** presents full 60 minute time traces for when the pump is on and milli-Q water is pumped in the channels and in **Figure S14B** the same measurement with the pump turned off.

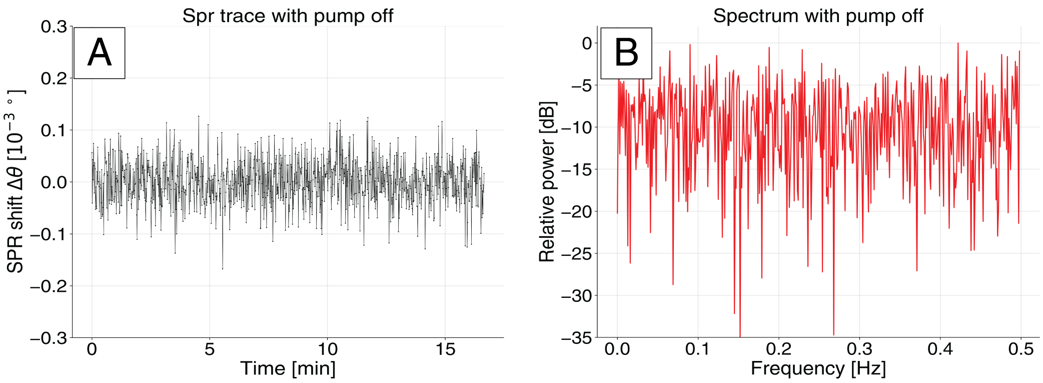

Figure S13: A) The raw time trace from the experiment while tracking the intensity resonance shift from water, $n = 1.333$, with the pump turned off. **B)** Fourier transform of the raw time trace. The background noise spectrum without the pump has a uniformly distributed power over all frequencies, i.e. white noise.

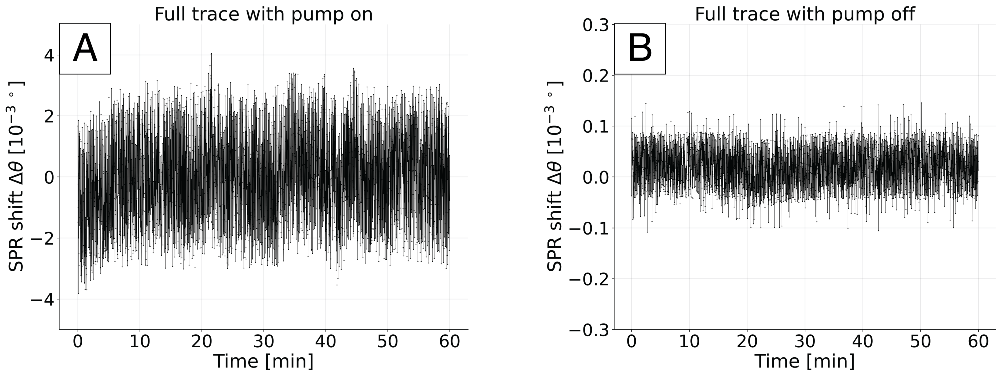

Figure S14: 60 minutes time traces with milli-Q water in the sensing channels. **A)** is the time trace when the pump is turned on. This trace is used to calculate the reported resolution and a 12 min section is presented in **Fig. 4D**. **B)** is the full time trace when the pump is turned off and there is no flow in the sensing channels.

8 miR-122 assay

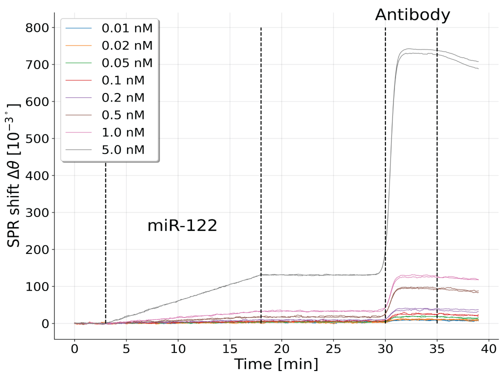

Figure S15: The full time traces from the miR-122 assay. From 0-3 min the Tris_{Mg} buffer is flown to get a baseline for the measurement, after which miR-122, diluted in Tris_{Mg}, binds to the sensor surface for 15 min, followed by Tris_{Mg} for another 12 min. Lastly at 30 min the antibody that binds to the RNA*DNA duplex is introduced.

The full time traces from the miR-122 assay are presented in **Fig. S15**. This is the same graph as in **Fig. 6A** but with two additional measurements with 5 nM concentration of miR-122 that were left out from the figure because of the large response, which makes the lower concentrations indistinguishable. The presented values for the specific direct detection of miR-122 are calculated by subtracting the response from unspecific binding in a reference channel, running in parallel with the presented miR-122 measurements. The reference channel has the same functionalization, but is not injected with any miR-122. The presented data response for the specific binding is taken by a linear fit to the response when miR-122 is flown, from 3-18 min, and extracting the final value, at $t = 18$ min, for the fitted response. The same fitting is performed for the response in the reference channel, and the final reported value for the specific binding is taken by subtracting the unspecific response in the reference channel. Time traces and the fitted lines are presented in **Fig. S16A** for the measurement with 1.0 nM concentration of miR-122.

The reported specific response from the antibody amplified response is calculated in the same manner. The response is taken as the average over 1 min after the antibody has saturated the surface at $t = 32$ min. Then the specific response is calculated by subtracting the response from the reference channel. In **Fig. S16B** the antibody amplified response with the performed averaging is presented for 1.0 nM concentration of miR-122.

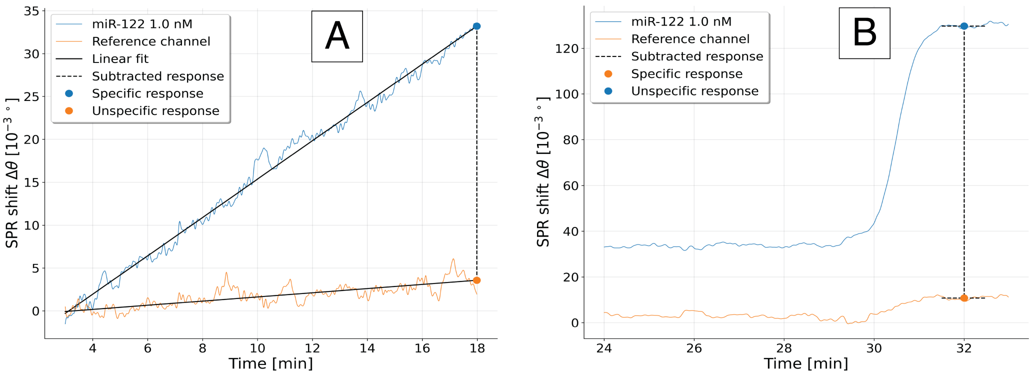

Figure S16: **A)** The sensor response from a channel with 1.0 nM of miR-122 together with the corresponding time trace for the reference channel. The reported specific binding rate is taken by subtracting the unspecific response in the reference channel. **B)** The antibody amplified response from the same measurement of 1.0 nM of miR-122. The specific response is found by subtracting of the response in the reference channel. The presented response is the average over 1 min around $t = 32$ min, as indicated in the figure.

9 Comparison of SPR instrumentation

Table S1: Comparison of the performance metrics sensitivity and resolution for SPR instrumentation, together with information on the excitation method, physical size and number of channels. The table only includes the sensitivity for devices that measures the angle shift of the SPR, for comparison with the presented device. ND - not disclosed.

**Instrument** *Company*	**Sensitivity**	**Resolution** [Δn]	SPR excitation	Size	# Channels
**State-of-the-art devices**					
Biacore 8k [7] *Cytavia*	ND	$< 10^{-6}$	Focused beam	Large	8
BioNavis-200 Navi [8] *BioNavis*	86.3 °/RIU	$1.15 \cdot 10^{-5}$	Sweeping collimated beam	Benchtop	2
**Compact SPR devices**					
Spreeta 2000 [9] *Texas Instruments*	82.3 °/RIU	$3.0 \cdot 10^{-6}$	Divergent beam	28 cm x 22 cm x 13 cm	8x3
SPRCD [10]	Measures wavelength shift	$2.0 \cdot 10^{-7}$	Grating coupling	15 cm x 15 cm x 15 cm	4
NanoSPR8 [11] *NanoSPR Instruments*	ND	$2.0 \cdot 10^{-5}$	Waveguide coupling	18 cm x 13 cm x 10 cm	8
P4SPR [12] *Affinite Instruments*	ND	$2.0 \cdot 10^{-7}$	Grating coupling	18 cm x 16 cm x 6 cm	4
**Lab Devices**					
SBC-SPR [13]	160 °/RIU	$1.0 \cdot 10^{-5}$	Focused beam	20 cm x 20 cm x 9 cm	1
Miniature fiber-optic SPR [14]	Measures wavelength shift	$2.2 \cdot 10^{-4}$	Fiber coupling	–	1
Smartphone fiber-optics SPR [15]	Measures intensity shift	$7.4 \cdot 10^{-5}$	Fiber coupling	12 cm x 6 cm x 2 cm	1
Smartphone grating-coupled SPR [16]	Measures intensity shift	$4.1 \cdot 10^{-5}$	Grating coupling	14 cm x 8 cm x 4 cm	1
Smartphone angle-resolved SPR [17]	91.0 °/RIU	$2.1 \cdot 10^{-6}$	Divergent beam	2 cm x 2 cm x 10 cm + smartphone	3
Metasurface integrated VCSEL SPR *This manuscript*	90.45 °/RIU	$4.9 \cdot 10^{-6}$ ($4.6 \cdot 10^{-7}$ without pump)	Divergent beam	2 cm x 2 cm x 1 cm + camera	3

References

[1] G. Ronald Hadley. "Effective index model for vertical-cavity surface-emitting lasers". en. In: *Optics Letters* 20.13 (July 1995), p. 1483.

[2] Mindaugas Juodėnas et al. "High-angle deflection of metagrating-integrated laser emission for high-contrast microscopy". en. In: *Light: Science & Applications* 12.1 (Oct. 2023), p. 251.

[3] Younes Ra'di, Dimitrios L. Sounas, and Andrea Alù. "Metagratings: Beyond the Limits of Graded Metasurfaces for Wave Front Control". en. In: *Physical Review Letters* 119.6 (Aug. 2017), p. 067404.

[4] Jiří Homola and Marek Piliarik. "Surface Plasmon Resonance (SPR) Sensors". In: *Surface Plasmon Resonance Based Sensors*. Ed. by Jiří Homola. Vol. 4. Berlin, Heidelberg: Springer Berlin Heidelberg, 2006, pp. 45–67.

[5] P. B. Johnson and R. W. Christy. "Optical Constants of the Noble Metals". en. In: *Physical Review B* 6.12 (Dec. 1972), pp. 4370–4379.

[6] Yoh Sano. "Optical anisotropy of bovine serum albumin". In: *Journal of Colloid and Interface Science* 124.2 (Aug. 1988), pp. 403–406.

[7] Cytiva. *Biacore 8k*. Sept. 7, 2025. URL: `https://www.cytivalifesciences.com/en/us/shop/protein-analysis/spr-label-free-analysis/`.

[8] Eleonora Macchia et al. "Plasmonic Single-Molecule Affinity Detection at 10e-20 Molar". en. In: *Advanced Materials* 37.9 (Sept. 2025), p. 2418610.

[9] Timothy M. Chinowsky et al. "Portable 24-analyte surface plasmon resonance instruments for rapid, versatile biodetection". In: *Biosensors and Bioelectronics* 22.9-10 (Apr. 2007), pp. 2268–2275.

[10] Marek Piliarik et al. "Compact multi-channel high-sensitivity biosensor based on spectroscopy of surface plasmons". In: San Jose, CA, Feb. 2009, p. 719212.

[11] NanoSPR Instruments. *NanoSPR8*. Sept. 7, 2025. URL: `https://nanospr.com/nanospr8-eight-channel-electrochemical-surface-plasmon-resonance-spectrometer/`.

[12] Affinite Instruments. *P4SPR*. Sept. 7, 2025. URL: `https://www.affiniteinstruments.com/p4spr-2-0`.

[13] Amanda K. L. Freitas and Leiva C. Oliveira. "A Portable Surface Plasmons Resonance Sensor Based on Single-Board Computer". In: *IEEE Transactions on Instrumentation and Measurement* 72 (2023), pp. 1–9.

[14] Yun Peng et al. "Miniature fiber optic SPR high sensitivity humidity sensor based on coated polyvinyl alcohol film". en. In: *Optical Fiber Technology* 87 (Oct. 2024), p. 103934.

[15] Samuel S. Hinman, Kristy S. McKeating, and Quan Cheng. "Surface Plasmon Resonance: Material and Interface Design for Universal Accessibility". en. In: *Analytical Chemistry* 90.1 (Jan. 2018), pp. 19–39.

[16] Hasan Guner et al. "A smartphone based surface plasmon resonance imaging (SPRi) platform for on-site biodetection". In: *Sensors and Actuators B: Chemical* 239 (Feb. 2017), pp. 571–577.

[17] Pakorn Preechaburana et al. "Surface Plasmon Resonance Chemical Sensing on Cell Phones". In: *Angewandte Chemie International Edition* 51.46 (Nov. 2012), pp. 11585–11588.
